# Teodorico Borgognoni’s Formulary for Thirteenth Century Anesthetic Preparations

**DOI:** 10.3390/life13091913

**Published:** 2023-09-14

**Authors:** Valeria Cavalloro, Francesca Soddu, Sandro Baroni, Francesco Saverio Robustelli della Cuna, Eleonora Tavazzi, Emanuela Martino, Simona Collina

**Affiliations:** 1 Department of Earth and Environmental Sciences, University of Pavia, Via S. Epifanio 14, 27100 Pavia, Italy; valeria.cavalloro01@universitadipavia.it; 2 NBFC, National Biodiversity Future Center, 90133 Palermo, Italy; 3 Neuroimmunology Laboratory, IRCCS Mondino Foundation, Via Mondino 2, 27100 Pavia, Italy; francescasoddu27396@gmail.com; 4 Maimeri Foundation, Corso Cristoforo Colombo 15, 20144 Milano, Italy; sandro.baroni@yahoo.it; 5 Department of Drug Sciences, University of Pavia, Viale Taramelli 12, 27100 Pavia, Italy; fsaveriorobustelli@unipv.it (F.S.R.d.C.); simona.collina@unipv.it (S.C.); 6 Multiple Sclerosis Centre, IRCCS Mondino Foundation, Via Mondino 2, 27100 Pavia, Italy; eleonora.tavazzi@mondino.it

**Keywords:** Teodorico Borgognoni, ethnobotany, anesthesia

## Abstract

Teodorico Borgognoni was born in Lucca in 1205 and was appointed bishop of Bitonto and Cervia in 1262 and 1270. Following his father, he learned the art of surgery and collected relevant recipes in his most important work, entitled *Cyrurgia seu filia principis*. Among the disciplines reported in this work, the most interesting and innovative is anesthesia. The recipes in this field contribute to Borbognoni’s consideration as the forerunner of modern anesthesia. Such recipes have been reported in other manuscripts from the Middle Ages, like Manuscript No. 1939. In the present work, we investigate the traditional preparations handed down in this manuscript, focusing on type of preparation and botanical ingredients. The results highlight that exploited ingredients can be divided into three groups: the first comprises plants already known for their narcotic effects, the second includes ingredients acting as an adjuvant for absorption or reducing the metabolism, and the last group includes ingredients not associated with biological activity to explain their presence in anesthetic recipes. This third group is of particular interest for future biological investigations. Our goal is to rekindle attention to the work of Teodorico Borgognoni on traditional preparation for anesthetic purposes: a topic often underestimated by ethnobotanical surveys.

## 1. Introduction

Teodorico Borgognoni (Lucca, 1205-Bologna, 1298) became part of the Dominican Order of the San Domenico Convent in Bologna at the age of 19 and was appointed bishop of Bitonto and Cervia in 1262 and 1270, respectively. During the same years, following his father’s footsteps, he also became a medical doctor with a special interest in surgery and, more specifically, in the treatment of skull injuries, breast cancer, and gum disease. Over the years, Borgognoni became known for his alternative approaches to the treatment of several medical conditions. For example, he used warm wine washes followed by a simple bandage to treat wounds, realizing the importance of keeping the wound clean and protected [1]. This innovative approach differed from the traditionally recommended procedure suggested by the Salerno Medical School, which considered pus as the best natural treatment (“*pus bonum et laudabile*”). Borgogni also focused his attention on the importance of a proper dietary regimen for convalescents, recommending a rigorous diet rich in foods considered capable of regenerating blood, such as meat, while prohibiting drinking with the exception of wine [2].

All the recipes collected and adopted by Borgognoni were written down by the bishop himself in his most important work, the treatise *Cyrurgia seu filia principis*. It consists of four volumes, each one dedicated to the treatment of different diseases: wounds are the subject of the first book, fractures and dislocations in the second, fistulas, gangrene, hernias, scabies, and leprosy in the third, and other diseases like headaches, paralysis, epilepsy, gout, and impaired vision in the fourth one.

Among Borgognoni’s scientific interests, anesthesiology was the main one.

In previous centuries, patients were sedated before surgery with only few alternative methods, including the use of plants or physical maneuvers, which were sometimes dangerous. In 50 B.C., the Greek physician Dioscorides, the first to use the term anesthesia (ἀναισϑησία or anaisthēsía, loss of sensitivity), described the narcotic effects of mandrake (*Mandragora officinarum* L.) in *De materia medica*. He claimed that mandrake decoctions in wine could be used to make patients insensitive to incisions and cauterizations [3]. Mandrake was also used with the same aim in other cultures, as evidenced by Egyptian and Jewish archaeological findings and described in the Old Testament, but it was also almost the only alternative available at that time. During these years, other cultures also developed new anesthetic methods, like in Aiurvedic traditional medicine, where Sangyaharana is the Sanskrit term for reversible loss of sense. For example, ancient Indian medical texts, e.g., Sushruta Samhita (400 B.C.), advocate the use of madya-wine, the incense of *Cannabis sativa* (Indian hemp), various herbs like *Withania somnifera* (Ashwagandha), *Bacopa monnieri* (Brahmi), *Grewia asiatica* (Parsikyavani), and others as premedicates to achieve tranquility and hypnosis before operation [4].

Borgognoni was one of the first surgeons to adopt *Spongia Somnifera* as an anesthetic; a sponge dipped in a mixture of extracts obtained from different plants like the already mentioned Madrake, but also *Solanum nigrum*, *Hyoscyamus niger*, *Cicuta minor*, and *Datura stramonium*. *Spongia Somnifera* is then kept in hot water for about 1 h and finally pressed against the patient’s nostrils and mouth [5]. Moreover, he also recommended the use of alternative extracts and mixtures, each with specific indications. Borgognoni collected a series of recipes to anesthetize patients in *Cyrurgia seu filia Principis*, exploiting plants developed for *Spongia Somnifera* and others like *Solanum nigrum*, while also resuming and theorizing methods already tested by his own father, leading several authors to question the originality of his work. Despite this criticism, anesthetic remedies by Borgognoni have a high impact on society, and some of his recipes have been reported in other fundamental works of the Middle Ages, in which Borgognoni is cited as the father of modern anesthesia.

One of the most important works recording his knowledge is Manuscript No. 1939, nowadays kept in the state library of Lucca and entitled *Ars sive doctrina Hermetis sapientissimi phylosophi et catholici christiani de transmutatione omnium mettalorum*. This work is a miscellaneous collection of recipes and treatises related to different topics: medicine, alchemy, personal care, art, and home remedies. Moreover, in the same manuscript, nine of Borgognoni’s recipes for anesthetic purposes are reported.

In the present work, we discuss the traditional preparations reported in Manuscript No. 1939, with a particular focus on the plants exploited in these recipes. Our ultimate goal is to rekindle attention to the work by Teodorico Borgognoni and, in particular, to the traditional preparations of anesthetics. Thus, the study of traditional preparations could identify active herbal extracts or pure metabolites, which are also useful in modern medicine. Despite the well-known positive results obtained when pursuing this approach, traditional recipes exploited in the anesthetic field are often underestimated by ethnobotanical surveys. This statement makes the study of Borgognoni’s work important to deepen knowledge in this field.

## 2. Results and Discussion

### 2.1. Stylistic Analysis of Manuscript No. 1939

**Figure 1:**
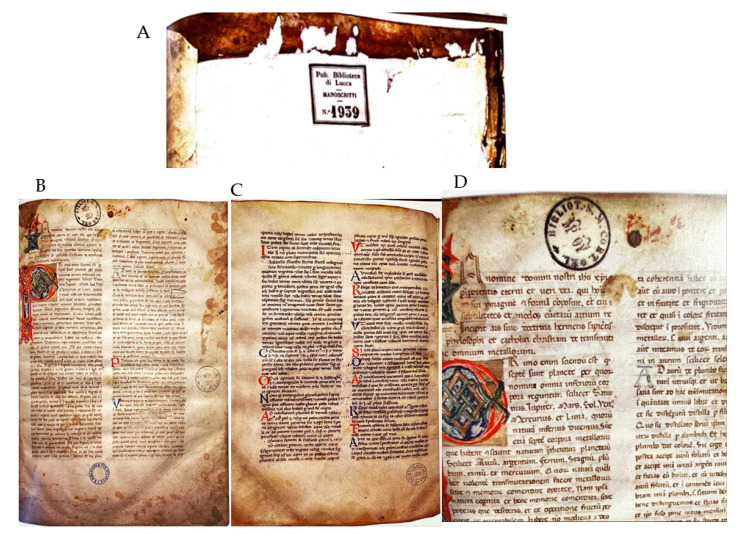
(**A**) Label present in the front flyleaf with the indication “Pub. Library of Lucca, Manuscripts, N°1939”; (**B**) Public Library of Lucca stamps; (**C**) Government Library stamps; and (**D**) Library of Santa Maria in Corteorlandini stamps. Pictures: Baroni-Segre snc Photographic Archive, Milan (IT).

Manuscript No. 1939 dates to the 14th century; it is located in the state library of Lucca, and it is written on parchment. A preliminary stylistic analysis of the manuscript suggests that its cover was probably added later. Indeed, the manuscript has a parchment binding decorated with the filigree letter “d” placed on the front cover with a style dated between the 15th and 16th centuries. The Greek stitching seems to be original, made of ox sinew. In the lower part of the spine, a paper label is visible showing the inscription “Government Library of Lucca, Manuscripts 1939”, while a second label is present in the front flyleaf with the indication “Pub. Library of Lucca, Manuscripts, N°1939” (Figure 1A). Moreover, inside the manuscript, there are stamps belonging to the Public Library of Lucca (Figure 1B), where two are from the Government Library (Figure 1C) and three from the Library of Santa Maria in Corteorlandini (Figure 1D).

As noticeable from Figure 1, the pages are organized in two parallel columns composed of fifty lines each. The binding was realized with the tip of a pen, and the ink color was brown. The text appears homogenous, mainly in the Gothic style, suggesting that it was written by a single scribe.

All the recipes, 2143 in total, of which 169 are now lost, were written in Latin except for two recipes in French, with some vernacular writings on the first and last pages.

### 2.2. Recipes from Borgognoni

These 2143 recipes cover topics from medicine and alchemy to home remedies. The recipes and treatises about medical nature are the most represented ones, followed by the alchemy recipes detailing home remedies, body care, and the ones related to artistic techniques.

Of particular interest for the present work, nine recipes that can be attributed to Borgognoni have been found in Manuscript No. 1939 on pages 4 verso and 4 rectos (4v and 4r). These are medical prescriptions that date between the end of the 13th century and the beginning of the 14th century, with instructions for the preparation of soporific and anesthetic potions. These preparations were generally adopted in surgical practices, even if the different titles might also suggest other purposes. The main characteristics (title, preparation, and ingredients) of these recipes are summarized in Table 1.

In the next paragraphs of the present work, the main ingredients and kinds of preparations reported in the recipes by Borgognoni are discussed.

### 2.3. Natural Matrices Reported in Borgognoni Recipes

In this section, we report and analyze the botanical ingredients used to prepare Borgognoni’s recipes.

#### 2.3.1. *Papaver* spp.

*Papaver* spp. are herbaceous plants belonging to the Papaveraceae family, which are native to the temperate and subtropical Northern Hemisphere and South Africa.

In Borgognoni’s recipes, two species belonging to the genus *Papaver* are reported: *P. somniferum* L. and *P. rhoeas* L., whereas *P. rhoeas* (mentioned as the red poppy) is cited only in one recipe (#1) and only for its seeds. Modern studies mainly focus on the aerial parts of this plant, and its hydroalcoholic extract has already been associated with activity in the central nervous system (CNS) [6,7,8]. The presence of *P. rhoeas* seeds in recipe #1 could suggest the necessity for a deeper investigation of its biological activity.

**Figure 2:**
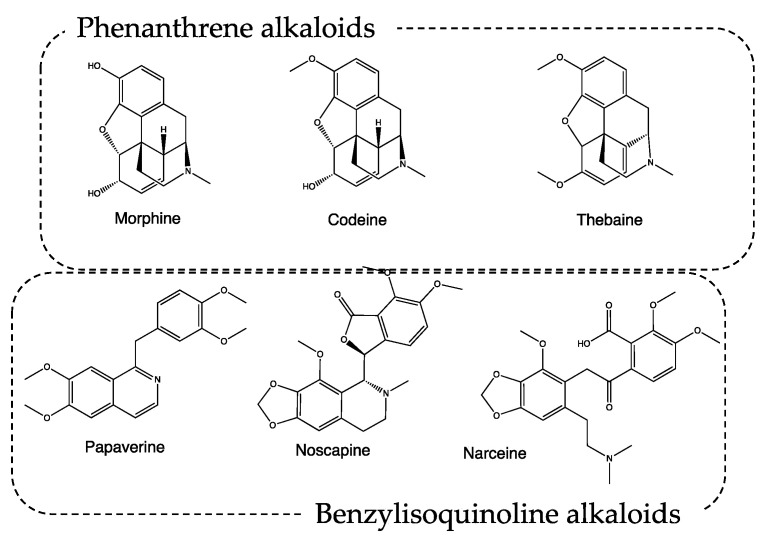
Chemical structure of alkaloids from *Papaver* spp.

Conversely, *P. somniferum*, mentioned both as the white poppy, probably for var. *album* (recipes #1, 2, 3, 6, 7, 8), and black poppy, probably for var. *nigrum* (recipes #2, 8) is well known for its narcotic effect. It was called the opium poppy, and it was used by ancient Egyptians, Persians, Greeks, and Romans for both medical and dietary purposes [9]. The name derives from the Latin “*somnus fero*”, and “which makes you sleep”, and is indicative of plant applications. The most active plant component is opium (mentioned in recipes #1, 6, 7), which is obtained by cutting the immature capsules of *P. somniferum* and collecting the latex that oozes after drying in the sun [10]. The phytocomplex contains tannins, resins, mineral salts, polyphenols, and alkaloids, which can be classified as phenanthrene and benzylisoquinoline alkaloids. Among the metabolites classified as phenanthrene alkaloids, the most important ones are morphine, codeine, and thebaine, endowed with analgesic–narcotic activity. The benzylisoquinoline alkaloids papaverine, noscapine, and narceine are instead characterized by a pure muscle relaxant action (Figure 2). The action induced by the aforementioned secondary metabolites can be favored by the presence of tannins, which slow down the gastric metabolism of the active alkaloids by inhibiting digestive enzymes.

Borgognoni also used the seeds (recipes #1, 2, 8) and leaves (recipe #3) of *P. somniferum*. Both these matrices have been recently analyzed, and these results have highlighted that both contain morphine and thebaine, while leaves also contain codeine [11]. These recent outcomes further validated the effectiveness of Borgognoni’s treatments.

#### 2.3.2. *Hyoscyamus niger* L.

*Hyoscyamus niger* L. is an annual/biannual herbaceous plant belonging to the Solanaceae family and the native temperate of Eurasia, North-Western Africa. Borgognoni cites this plant as Henbane in four different recipes (#4, 5, 6, 8).

**Figure 3:**
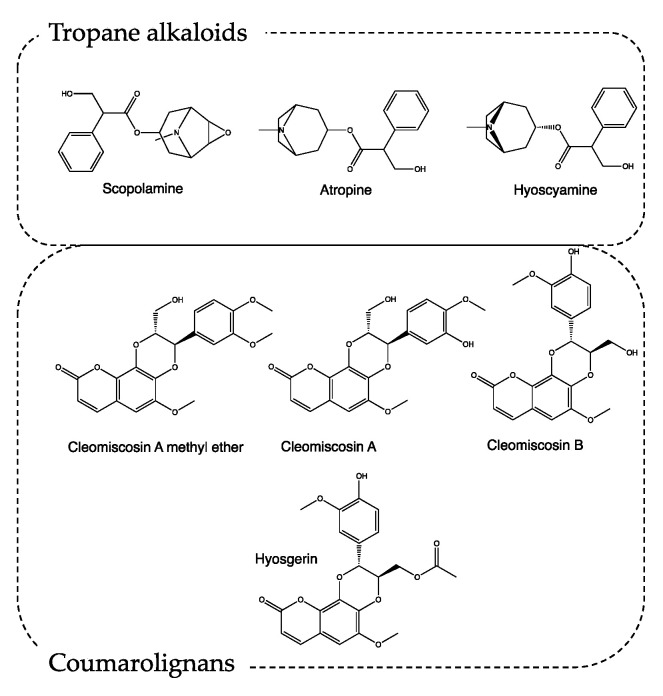
Chemical structure of secondary metabolites from *Hyoscyamus niger*.

The most interesting secondary metabolites produced by *H. niger* are tropane alkaloids such as scopolamine, atropine, and hyoscyamine (Figure 3) [12]. The tropane nucleus alkaloids act as antagonists of muscarinic receptors, promoting parasympatholytic and neuro-depressive activity. Particularly, atropine, if administered in doses between 0.5 and 2 mg, can induce drowsiness and cause a loss of concentration and spatial misperception. At higher doses, it can induce lethargy, depersonalization, and hallucinations [13,14]. Scopolamine shows a similar profile, with a 10-fold more powerful activity [15].

In recipe #8, Borgognoni exploits only *H. niger* seeds. This matrix is particularly rich in secondary metabolites, like tropane alkaloids, lignans, lignamides, coumarolignamides, flavonoids, withanolides, and saponins. Recently, the methanolic extraction of *H. niger* seeds resulted in being able to increase the latency time of the pain response acting both on the central and peripheric nervous systems [16,17]. Moreover, the characterization of this methanolic extract highlights the presence of coumarolignans, whose main representatives are cleomiscosin A methyl ether, hyosgerin, cleomiscosin A, and cleomiscosin B (Figure 3). According to the literature, this class of secondary metabolites, particularly clemiscosin A and B, have anti-inflammatory activity [18].

#### 2.3.3. *Mandragora officinarum* L.

*Mandragora officinarum* L. is a perennial plant belonging to the *Solanaceae* family and was originally found in a large geographical area extending from Northern Italy to the Northern-Western Balkan peninsula. As for *H. niger*, mandrake has also been mentioned by Borgognoni in four different recipes: #4, 5, 6, and 7.

As mentioned in the introduction section, mandrake was the first plant utilized for anesthetic purposes in different cultures (Greek, Egyptian, and Jewish) [17,18,19]. In *Naturalis historia*, Pliny the Elder (23–79 A.D.) stated that this mandrake-based drink was an excellent antidote for snake bites, but it was also useful before surgery (*ante sectiones punctionesque*). The use of mandrake as an anesthetic remedy entered medieval practice thanks to Isidore of Seville (560–636 A.D.), who, in an encyclopedic work of 20 books, described its characteristics and narcotic use [20]. Mandrake juice is also used in the preparation of already mentioned *Spongia Somnifera* or soporific sponges.

Thanks to modern outcomes, the fruits and roots of *M. officinarum* have now been characterized, and the results highlight that they contain tropane alkaloids, such as the already-mentioned atropine, hyoscyamine, and scopolamine responsible for healing, hallucinogenic and toxic activities [21,22]. As stated before, the tropane alkaloids act as antagonists of the muscarinic receptors.

#### 2.3.4. *Lolium perenne* L.

*Lolium perenne* L. is a biennal/perennial plant belonging to the *Poaceae* family, which is native to Macaronesia, Northern Africa, and Europe, extending to Siberia and the Himalayas. *L. perenne*, as has been mentioned already by Borgognoni in recipes #1 and 8. In this last recipe, Borgognoni specified how only Ryegrass seeds are exploited: a matrix recently associated with the production of proanthocyanidins, metabolites with antioxidant activity [23].

**Figure 4:**
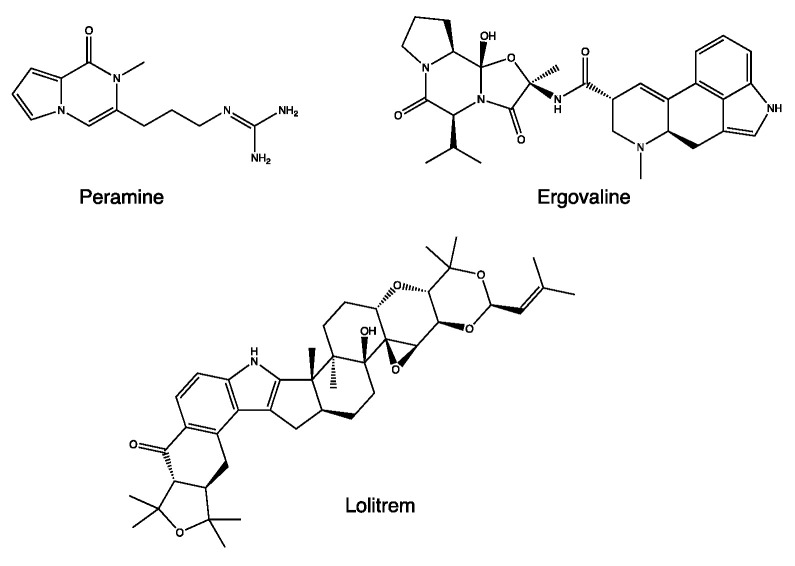
Chemical structure of secondary metabolites from *Lolium perenne*.

The properties of *L. perenne* are linked to the presence of alkaloids produced by the endophytic fungus *Neotyphodium coenophialum*, such as peramine, ergovaline, and loritrem B (Figure 4) [24]. Ergovaline is an ergopeptide belonging to the ergot alkaloid family. It can inhibit the activity of the vesicular glutamate transporter 1 (VGLUT) through a non-competitive mechanism, resulting in a reduction in glutamatergic transmission and leading to a reduction in vigilance [25]. On the other hand, loritrem B is a “tremogenic” neurotoxin, an antagonist of high-conductance calcium-dependent potassium channels. It causes tremors and ataxia and reduces orientation in space [26]. These two metabolites are associated with high toxicity, higher than peramine, with the other main metabolite of *N. coenophialum*.

#### 2.3.5. *Anagyris foetida* L.

*Anagyris foetida* L. is a plant belonging to the Fabaceae family, native to Mediterranean regions and the Arabian Peninsula. It has been cited in two different recipes by Borgognoni, particularly #1 and 2.

**Figure 5:**
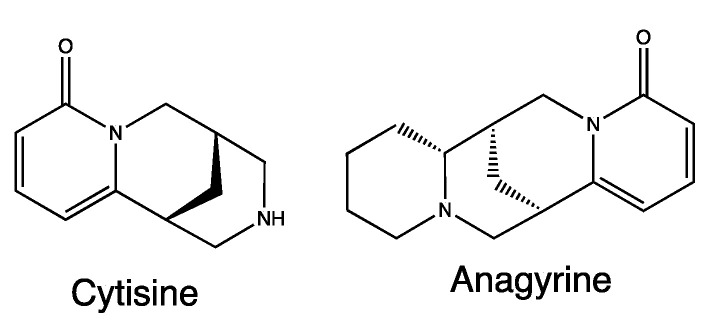
Chemical structure of secondary metabolites from *Anagyris foetida*.

*A. foetida* extracts mainly contain quinolizidine alkaloids such as cytisine and anagyrine (Figure 5) [27], metabolites already associated with different pharmacological and toxicological properties, such as CNS depressant, hypotensive and hallucinogenic properties. The activity of these alkaloids is probably linked to the modulation of cholinergic receptors and the ion channels of Na^+^ and K^+^ [28].

#### 2.3.6. *Lactuca sativa* L.

*Lactuca sativa* L. is an annual/biannual herbaceous plant belonging to the Asteraceae family and is native to Western Asia. Borgognoni cites this plant, and particularly its seeds, in recipes #5 and 8.

This particularly natural matrix contains triterpenes, saponins, and phenols. Triterpenes have anti-inflammatory activity [29] as they inhibit the inducible enzymes nitric oxide synthase (iNOS) and cyclooxygenase 2 (COX-2), which are both involved in inflammatory process [30]. Saponins are also able to exert an anti-inflammatory action, inhibiting the already-mentioned enzymes and lipoxygenenase [31]. Studies conducted on mouse models have shown that lettuce seed extract has an antinociceptive action. Although its interaction with the opioid receptors of triterpenes, saponins, and phenols has been excluded, the exact mechanism of its activity is not yet clear, and further studies should be performed.

#### 2.3.7. Other Plants

**Figure 6:**
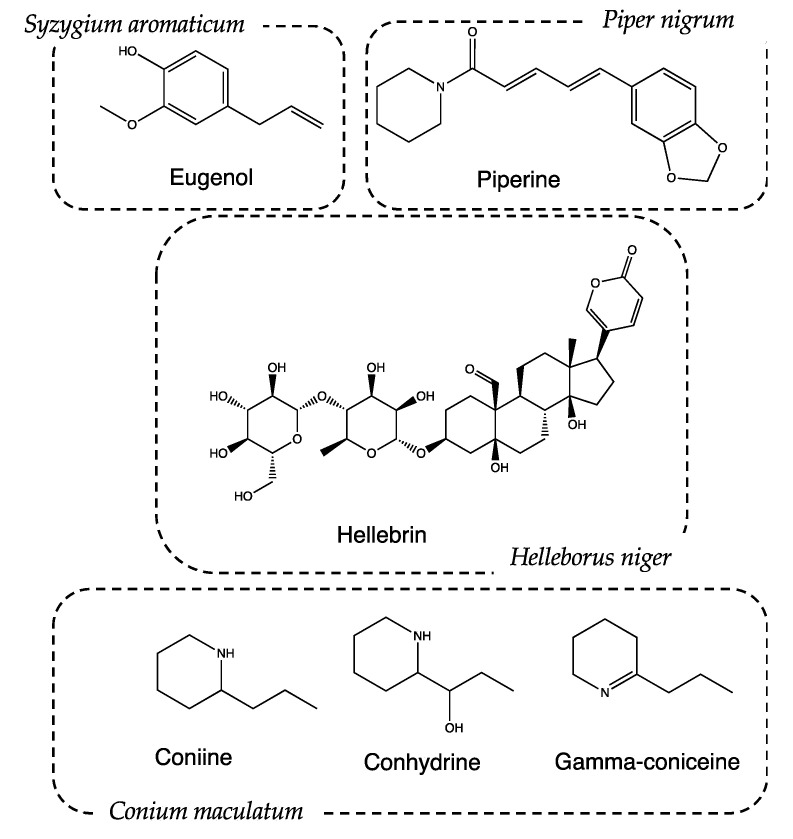
Chemical structure of main metabolites identified in the considered plants.

*Syzygium aromaticum* (L.) Merr. and L.M. Perry, (Recipe #2). The pharmacological and aromatic properties of this plant have been known since ancient times in China but were ignored for a long time by Greeks and Latins. In the Middle Ages, some types of cloves, called “gariophiles”, were also used for therapeutic purposes. The Medical School of Salerno considered them a panacea, effective in the treatment of mental fatigue or memory loss. Clove essential oil is characterized by the presence of eugenol (Figure 6), a metabolite with anesthetic activity [32], as it inhibits the conduction of voltage-gated sodium channels at the level of dental nerve endings [33]. Eugenol also has anti-inflammatory and analgesic activity, which are associated with the prevention of neutrophil/macrophage chemotaxis and the inhibition of the synthesis of inflammatory neurotransmitters, such as prostaglandins and leukotrienes. Furthermore, eugenol dimers have shown chemopreventive properties due to the inhibition of cytokine expression in macrophages [34].

*Aloe vera* (L.) Burm. F. (Recipe #2). Only yellow wood is mentioned as an ingredient for this plant. Interestingly, nowadays, the gel contained inside the leaves is part of a component that is most widely used for skin inflammation, cosmetic and food purposes. Recent work highlighted that the main metabolites produced by *A. vera* are anthraquinones, glucomannan, and acemannan [35].

*Verbascum thapsus* L. (Recipe #3). Only limited data are available regarding the biological activity of this plant. Recently, it was demonstrated that *V. thapsus* contains mucilages, saponins, and iridoid terpenes [36]. Saponins could facilitate the absorption of other substances, while mucilages absorb and retain water, supposedly contributing to the stability of the compound.

*Piper nigrum* L. (Recipe #4). Traditional uses of *P. nigrum* include the treatment of muscle pain and rheumatism [37,38]. The action is mainly given by the presence of piperine: a protoalkaloid extracted from its fruits (Figure 6). Piperine helps increase the bioavailability of drugs, presumably by increasing their absorption using alternating membrane lipids, intestinal enzymes, and the stimulation of the activity of leucine-amino-peptidase and glycyl-glycine-dipeptidase [39]. It is reasonable to assume that *P. nigrum* favors the absorption of active ingredients with a narcotic action, thus promoting patients’ sleep.

*Helleborus niger* L. (Recipe #5). *Helleborus niger*, commonly called poinsettia, is a highly toxic plant due to the presence of bufadienolides (cardioactive glycosides), such as helleborin, hellebrin and helleborein. They belong to the class of cardioactive glycosides and have a positive inotropic effect, increasing cardiac contractility. Hellebore was exploited in ancient times for its hallucinogenic properties, as reported by Stobeo [40]. The mentioned hallucinogenic activity is correlated to the presence of tropane alkaloids produced by the seeds of *H. niger*, particularly hyoscyamine and scopolamine. As previously assessed, these compounds cause drowsiness due to their anticholinergic activity [41].

*Armoracia rusticana* G. Gaertn., B. Mey and Scherb (Recipe #7). The roots of *A. rusticana* contain substances such as isothiocyanates and thioglycosides, organic acids, and fatty acids. Hydantoins and thioidantoins are also part of the phytocomplex, metabolites associated with anti-inflammatory, antitumor, and antimicrobial activities [42,43]. Based on recent scientific evidence, the reason for the presence of *A. rusticana* in recipe #7 needs further elucidation.

*Styrax officinalis* L. (Recipe #7). In recipe #7, the natural matrix is named styrax. This could be referred to as *Liquidambar styraciflua*, also known as American styrax, but this plant was only brought in Europe in the 17th century. Consistently, this plant was identified as *Styrax officinalis*. Tannins and triterpene saponins [44] can act in synergy with substances with narcotic activity, favoring their absorption. Moreover, a recent study highlighted the presence of a considerable amount of melatonin inside the leaves of *S. officinalis* [45]. Melatonin is a hormone produced by the pineal gland that acts on the hypothalamus, regulating the sleep–wake cycle, and has sedative effects as well [46].

*Portulaca orolacea* L. (Recipe #8). *P. orolacea* seeds contain tannins, phenols, and flavonoids [47]. Tannins can favor the activity of the active ingredients present within narcotic–sedative action, as they have an astringent and inhibitory activity on digestive enzymes [48].

*Cerinthe major* L./*Symphytum officinale* L. (Recipe #8). In recipe #8, the natural matrix is named major grass. This generic name may refer to either *Cerinthe major* or *Symphytum officinale*. This ambiguity derives from the Italian common names *erba vajola maggiore* (literally major vajola grass) and *Consolida Maggiore* (literally major consolida), respectively. In the first case, characterizing *Cerinthe major* seed oil, it is possible to identify the presence of tocopherols, which have antioxidant activity, and polyunsaturated fatty acids, which have anti-inflammatory activity [49,50,51]. In the second case, *Symphytum officinale* root extracts were traditionally considered pain-killer effective against inflammation associated with joints, bone, and muscle ailments. Recent works have already demonstrated a broad spectrum of bioactivities and characterized the main constituent of the phytocomplex [52]. Despite the Italian common name, many think that *C. major* is the true assignment and that biological activity and traditional uses might move its assignment to *S. officinale*.

*Conium maculatum* L. (only ingredient of recipe #9). *C. maculatum* is one of the most poisonous plants historically known: much of its fame is linked to the death of the famous Greek philosopher Socrates. The court of Athens voted for the death sentence, and the execution of the philosopher took place by means of a potion based on hemlock [53]. Despite its high toxicity, if administered in adequate doses, *C. maculatum* can be used to induce analgesia and depression in the CNS, inducing sleepiness. These effects are attributable to the presence of piperidine alkaloids such as conhydrin, gamma conicein, conmaculatin, and, in particular, coniine [54,55,56]. These substances have activities that are very similar to curare and nicotine, both at a peripheral and central level [57,58,59]. Coniine is an antagonist of nicotinic receptors (nAChRs) and determines the block of transmission through the superior ganglion and the neuromuscular junction [60]. Thanks to its interaction with nicotinic receptors, coniine carries out an antinociceptive action by hindering the transmission of the pain signal in the nervous system [61].

### 2.4. Anesthetic Preparations

All these ingredients mentioned and described so far are used in different preparations [62].

In detail, anesthetic preparations (Table 1) were mostly powders obtained by grinding different ingredients and then administrated as such (recipes #4 and 7) or mixed with honey (recipe #5). This powder can also be mixed with hot water and then applied on the forehead of patients (recipe #6). This latter route of administration is the mildest and least invasive and is particularly useful to induce sleep rather than preparing patients for surgery, as suggested by the title “Sleeping pill for the infirm who are unable to sleep”. Tinctures based on the use of wine as solvent are preparations called enolith (recipes #1 and 2). Enoliths were introduced by Hippocrates, who dressed wine with nutraceutical characteristics, thanks to the use of the same as a solvent to obtain the extraction of active ingredients from medicinal plants, producing a curative and considered medicinal elixir. In detail, dry natural matrices are macerated in red wine to promote their astringent activity, while the precipitation of alkaloids caused by tannins is avoided or liquors if the matrix of interest contains a high number of resins. Enoliths were widespread at one time but were then progressively abandoned mainly due to their limited shelf life. Finally, two other preparations were described by Borgognoni: stabilized alcoholate (recipe #3) and distillate (recipes #8 and 9). In the first case, this preparation consisted of putting the fresh natural matrix in boiling alcohol, achieving both metabolite extraction and enzyme denaturalization. In such a way, a more stable preparation could be obtained. Conversely, the distillate was obtained by putting in contact vapors with a natural matrix and then condensing them.

## 3. Materials and Methods

**Figure 7:**
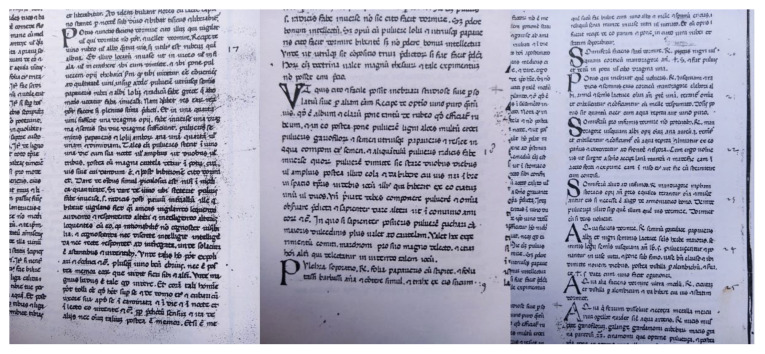
Original recipes by Borgognoni reported in Manuscript No. 1939.

The source of the present work is Manuscript No. 1939 The recipes considered are reported in pages 4v and 4r (Figure 7) and are hereunder resumed in English.

Recipe 1: Potion and anointing that puts one who is awake or who cannot sleep and wants to sleep to sleep. 

The first recipe recommends placing wine, preferably red, into a random container and adding the powder of opium, red and white poppy seeds, ryegrass, and stinking bean trefoil. The suspension so obtained should be left from 24 to 72 h before use. Rossignoli states that high doses of this treatment could be very dangerous but allow the subject to fall asleep very quickly. In the second part of this recipe, a simpler preparation is described, with the powder of stinking bean trefoil roots placed in wine. After the administration of this second treatment, the subject stays alert but is unable to see or interact with their environment. The recipe also reports that this second treatment is exploited by wives against their husbands or by thieves to make the victim harmless. Therefore, the author suggests the doctor keep the ingredients secret.

Recipe 2: How quickly and easily a scholar can be intoxicated, either for amusement or for some other cause.

This recipe indicates taking pure white wine and mixing it with red wine and next adding powder of aloe yellow wood, carnations, and poppy seeds. Simultaneously, it recommends putting seeds and stinking bean trefoil powder in water and waiting for two days. Finally, the subject can be made to drink. Sugar may be added to improve the flavor.

Recipe 3: Nice soporiferation.

This recipe recommends powdering fresh poppy leaves and aerial parts of Common Mullein, taking the juice, and boiling it with white wine and honey. The mixture must be stored in a glass and administrated by diluting a small aliquot in red wine.

Recipe 4: A sleeping pill that immediately puts you to sleep.

This recipe recommends powdering black pepper, henbane, and mandrake zest powder. This solid mixture should be added to drink and food.

Recipe 5: A potion that intoxicates whoever you want.

This recipe recommends gridding together henbane seeds and roots, mandrake zest, white hellebore and lettuce seeds. The obtained powder must be sieved, mixed with honey, and administrated with water and wine.

Recipe 6: Sleeping pill for the infirm who are unable to sleep.

This recipe recommends gridding together and sieving mandrake, white henbane, and opium. The powder so obtained must be packaged with warm water and put on the forehead and temples.

Recipe 7: Another sleeping pill for the sick.

This recipe recommends gridding together mandrake, horseradish, styrax, opium, and, if necessary, adding ammonium chloride. The powder, so obtained, must be administrated as it is to the subject.

Recipe 8: Water that makes you sleep.

This recipe recommends gridding together purslane seeds, black and white poppy seeds, lettuce seeds, grass seeds, ryegrass seeds, and henbane seeds. The mixture must be transferred in a glass vase and sealed under manure for nine days. Finally, the mixture is distillated and administered to the subject.

Recipe 9: More water that makes you sleep excessively.

This recipe recommends distilled hemlock and giving it to the subject to drink.

## 4. Conclusions

In the present work, recipes for anesthetic purposes reported in Manuscript No. 1939 have been analyzed. They were developed by Teodorico Borgognoni, a bishop who lived in the 13th century and is considered to be the father of modern anesthesia. The herbal ingredients used for recipe preparations can be divided into three groups based on their activity. The first group of plants comprises *Papaverum* spp., *Hyoscyamus niger*, *Mandragora officinarum*, *Lolium perenne*, *Anagyris foetida*, *Syzygium aromaticum*, *Helleborus niger*, and *Conium malculatum*, all associated with narcotic or analgesic effects. The second group includes *Verbascum thapsus*, *Piper nigrum*, *Styrax officinalis*, and *Portulaca orolacea*, all ingredients acting as an adjuvant for absorption or reducing metabolism. The last group includes four different ingredients that are not associated with a specific biological activity, which is useful to explain their presence in anesthetic recipes. This is the case for *Lactuca sativa*, *Aloe vera*, *Armoracia rusticana*, and *Cerinthe major*. Future research is needed to validate their use in preparations. As can be noticed by the structures of active metabolites identified in the herbal ingredients exploited, the most represented class is alkaloids. Thus, despite alkaloids often being associated with severe side effects, they are also often associated with therapeutic activity. Consistently, administrated doses should allow a balance between therapeutic and side effects. Unfortunately, neither doses nor side effects are mentioned in the analyzed recipes.

In the recipes, natural matrices are treated, and four different preparations are obtained: sometimes from simple powder, which is used as such, and sometimes mixed with wine or honey to create liquid preparations like enolith, stabilized alcoholate, or distillates.

To conclude, the study of old Manuscript No. 1939 allows us to understand the ancient use of herbal remedies and can explain the rationale behind it. Nevertheless, some points remain unexplained, and additional studies are needed to correlate the plant material used to reach an anesthetic effect.

Reading an old manuscript may also be a source of inspiration for future scientific work.

## Figures and Tables

**Table 1:** Title, preparation, and ingredients of the recipes by Borgognoni reported in Manuscript No 1939.

#	Title of the Recipes	Kind of Preparation	Ingredients
1	Potion and anointing that puts one who is awake or who cannot sleep and wants to sleep to sleep quickly.	Enolith	*Papaver somniferum* L. & P. rhoeas L. seeds *Lolium perenne* L. ^1^ *Anagyris foetida* L. roots
2	How quickly and easily a scholar can be intoxicated, either for amusement or for some other cause	Enolith	*Aloe vera* (L.) Burm.f. ^1^ *Syzygium aromaticum* (L.) Merr. & L.M.Perry ^1^ *Papaver somniferum* var. *album* & var. *nigrum* seeds *Anagyris foetida* L. ^1^
3	Nice soporiferation	Stabilized alcoholate	*Papaver somniferum* L. leaves *Verbascum thapsus* L. ^1^
4	Sleeping pill that immediately puts you to sleep	Powder	*Piper nigrum* L. ^1^ *Hyoscyamus niger* L. ^1^ *Mandragora officinarum* L. ^1^
5	Potion that intoxicates whoever you want	Powder mixed with honey	*Hyoscyamus niger* L. seeds and roots *Mandragora officinarum* L. peel *Helleborus niger* L. ^1^ *Lactuca sativa* L. seeds
6	Sleeping pill for the infirm who are unable to sleep	Powder mixed with hot water and applied on forehead	*Mandragora officinarum* L. ^1^ *Hyoscyamus niger* L. ^1^ *Papaver somniferum* L. ^1^
7	Another sleeping pill for the sick	Powder	*Mandragora officinarum* L. ^1^ *Armoracia rusticana* G. Gaertn., B. Mey & Scherb ^1^ *Styrax officinalis* L. ^1^ *Papaver somniferum* L. ^1^ *Ammonium chloride*
8	Water that makes you sleep	Distillate	*Portulaca orolacea* L. seeds *Papaver somniferum* var. *album* & var. *nigrum* seeds *Lactuca sativa* L. seeds *Cerinthe major* L. ^1^ *Lolium perenne* L. seeds *Hyoscyamus niger* L. seeds
9	More water that makes you sleep excessively	Distillate	*Conium malculatum* L. ^1^

^1^ The part of the plant exploited is not specified.

## Data Availability

Not applicable.
